# Development of patient-reported outcome for adult spinal deformity: validation study

**DOI:** 10.1038/s41598-024-51783-4

**Published:** 2024-01-14

**Authors:** Takahito Fujimori, Yukitaka Nagamoto, Shota Takenaka, Takashi Kaito, Yuya Kanie, Yuichiro Ukon, Masayuki Furuya, Tomiya Matsumoto, Shinya Okuda, Motoki Iwasaki, Seiji Okada

**Affiliations:** 1 Department of Orthopedic Surgery, Graduate School of Medicine, Osaka University, 2-2 Yamadaoka, Suita, Osaka 565-0871 Japan; 2 Department of Orthopedic Surgery, Osaka Rosai Hospital, Sakai, Osaka Japan; 3 Department of Orthopedic Surgery, Hoshigaoka Medical Center, Hirakata, Osaka Japan

**Keywords:** Quality of life, Health care, Medical research, Outcomes research

## Abstract

Adult spinal deformity (ASD) is a complex condition that combines scoliosis, kyphosis, pain, and postoperative range of motion limitation. The lack of a scale that can successfully capture this complex condition is a clinical challenge. We aimed to develop a disease-specific scale for ASD. The study included 106 patients (mean age; 68 years, 89 women) with ASD. We selected 29 questions that could be useful in assessing ASD and asked the patients to answer them. The factor analysis found two factors: the main symptom and the collateral symptom. The main symptom consisted of 10 questions and assessed activity of daily living (ADL), pain, and appearance. The collateral symptom consisted of five questions to assess ADL due to range of motion limitation. Cronbach’s alpha was 0.90 and 0.84, respectively. The Spearman’s correlation coefficient between the change of main symptom and satisfaction was 0.48 (p < 0.001). The effect size of Cohen’s d for comparison between preoperative and postoperative scores was 1.09 in the main symptom and 0.65 in the collateral symptom. In conclusion, we have developed a validated disease-specific scale for ASD that can simultaneously evaluate the benefits and limitations of ASD surgery with enough responsiveness in clinical practice.

## Introduction

Long fusion surgery for adult spinal deformity (ASD), performed only in a limited number of centers more than a decade ago, has rapidly spread and is now a standard and widely performed procedure^1^. ASD surgery was primarily performed for de novo scoliosis in the early days. Later, ASD became a broad disease concept that included sagittal imbalance as a surgical target. Thus, although ASD has complex conditions, patients with symptoms that warrant surgical treatment should have specific common problems.

**Figure 1:**
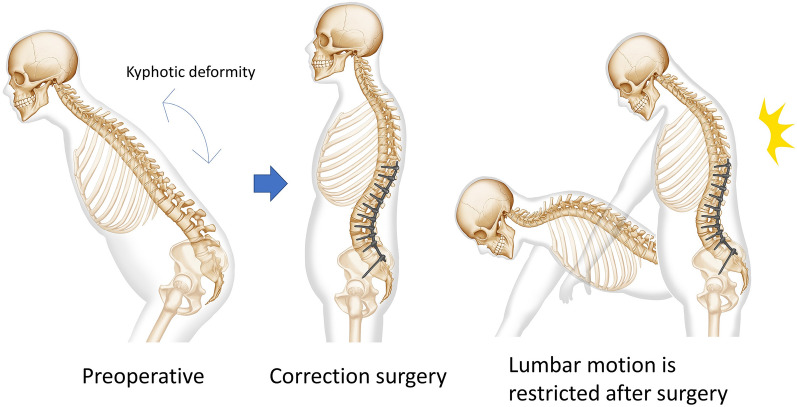
Schematic of changes in a typical long fusion surgery. Preoperatively, the patient cannot maintain posture due to kyphotic deformity. Postoperatively, the patient can maintain posture, but has limited range of motion.

The Scoliosis Research Society-22 Patient Questionnaire (SRS-22) is a standard questionnaire used to evaluate the treatment of scoliosis^2^. The SRS-22 is sometimes used to assess ASD as well, because no ASD-specific scale currently exists. However, the questions in the SRS-22 were designed primarily for adolescent idiopathic scoliosis (AIS). AIS and ASD have different ages of onset, various pathologies, and main complaints. In addition, in AIS, the lowest end of fixation is usually more proximal than L3, whereas, in ASD, the level of fixation often includes the pelvis, which is often accompanied by postoperative mobility restrictions^3,4^ (Fig. 1). Recently, Hart et al. developed the lumbar stiffness disability index to evaluate the limitation of motion of the spine due to long fusion surgery^5^. They called the restriction for activities of daily living (ADL) due to long fusion the collateral outcome. There is a trade-off relationship, so to speak, between improving pain due to fusion and restriction of range of motion. This trade-off is considered to be well established if the patient’s needs are met^6^. Thus, ASD presents a unique condition among spinal disorders that has elements of scoliosis but also kyphosis, as well as pain and limited postoperative range of motion. Although surgery for ASD is becoming more widespread, some researchers are concerned about the cost of the procedure and the high complication rate^7^. Conversely, conservative treatment of ASD includes medication, orthotics, Nordic walking canes, and walkers. These conservative treatments have the advantage of being less risky and less expensive than surgery and do not cause a postoperative range of motion limitations. However, conservative treatment could be less effective with respect to improving posture and pain. Furthermore, the use of a cane may be inconvenient for household activities because both hands are occupied when walking^8^. Currently, there is no HR-PRO that evaluates these life inconveniences from the perspective of ASD patients.

Therefore, we thought that a specific scale was needed to evaluate ASD. This study aimed to create a disease-specific patient-reported outcome measure (PROM) for ASD.

## Methods

### Patients

This study was a multicenter, self-report questionnaire survey conducted at two spine centers. In total, 106 patients were included: 97 patients who underwent long fusion surgery between 2007 and 2020 and nine patients who were undergoing conservative treatment and considering surgery for spinal deformity. The conservative patients had spinal deformities but preferred conservative treatment because their clinical symptoms were milder than those of the operative patients. A questionnaire consisting of 29 questions was mailed to these patients, and they were asked to complete and return it. Patients who had undergone surgery were asked to answer both preoperative and postoperative conditions. Conservatively treated patients were asked to answer questions about their current condition. A five-point satisfaction rating scale for surgery and Short-Form-8 (the physical component summary; PCS, and the mental component summary; MCS) were enclosed for criterion-related validation.

**Figure 2:**
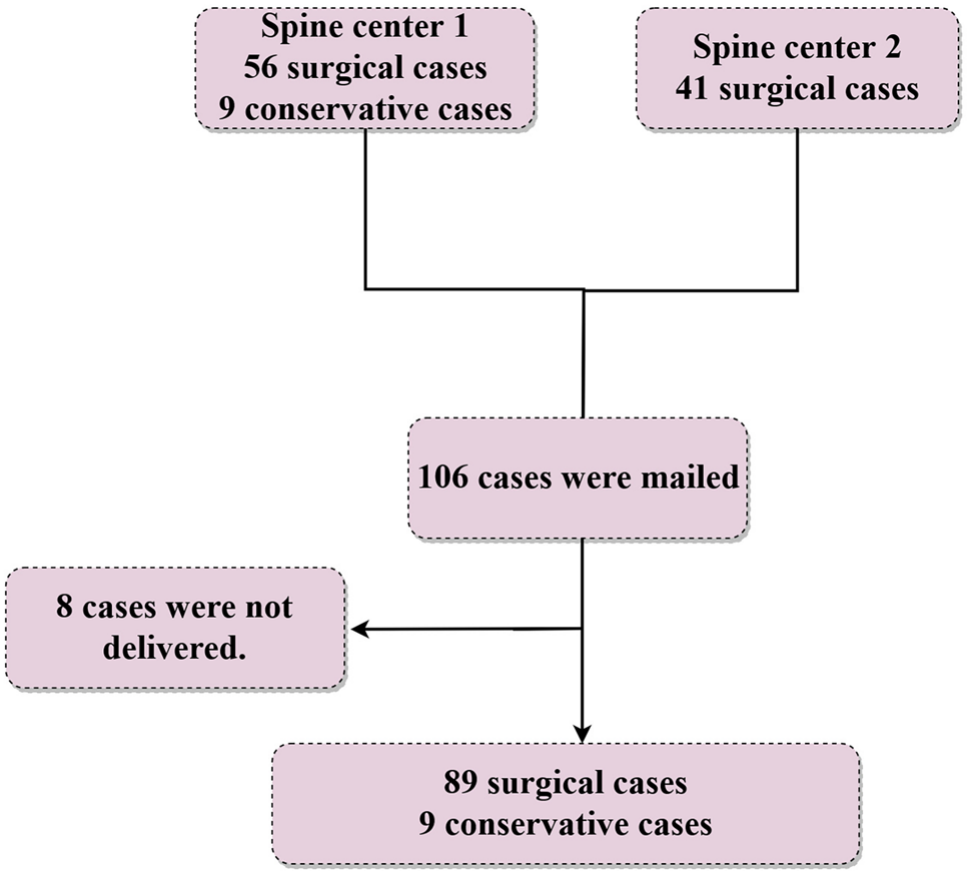
Patient flowchart.

Of the 106 patients, eight did not receive the mailing due to a change of address. The 98 patients (89 surgical patients) who responded were included in the study (Fig. 2). Long fusion was defined as the fusion of five or more vertebrae, including the lumbar spine. Fixation across the sacroiliac joint to the pelvis was counted as one vertebral segment. On imaging evaluation, all patients had a coronal plane Cobb angle > 30°, SVA > 40 mm, or pelvic tilt > 20°^9^.

### Selection of 29 questions

COnsensus-based Standards for the selection of health Measurement Instruments (COSMIN) aimed at improving the selection of PROM in research and clinical practice and some guidelines exist. We conducted this study in accordance with the COSMIN guidelines^10^. Content validity is the most important measurement property of PROM. It is the degree to which the content of an instrument is an adequate reflection of the construct to be measured. The criteria of content validity include the relevance, comprehensiveness, and comprehensibility of the PROM for the target population. We conducted a literature search to select questions relevant to ASD. We assumed that ADL, appearance, pain, mental health, and satisfaction would be the assessment items necessary to capture the disease concept of ASD^1,3,6,11–13^.

To develop the comprehensive questions, we reviewed a wide variety of existing questionnaires (Table 1), including Short-Form-36^14^, patient-reported outcomes measurement information system (PROMIS)^15^, Oswestry disability index (ODI)^16^, Roland–Morris questionnaire^17^, SRS-22^2^, Japanese Orthopedic association back pain evaluation questionnaire (JOABPEQ)^18^, Western Ontario and McMaster Universities Osteoarthritis Index (WOMAC)^19^, Knee Society Score^20^, Bath Ankylosing Spondylitis Functional Index (BASFI)^21^, Health Assessment Questionnaire (HAQ)^22^, pain disability assessment scale^23^, Zurich claudication questionnaire (ZCQ)^24^, EuroQol 5-dimensions 5-levels (EQ5D)^25^, lumbar stiffness disability index (LSDI)^5^, 25-question geriatric locomotive function scale (Locomo-25)^26^, gastroesophageal reflux disease questionnaire (GerdQ)^27^, and the Frequency Scale for the symptoms of gastroesophageal reflux disease (FSSG)^28^.

**Table 1:** Review list of the questionnaires.

	Abbreviation	Number of question items
The 36-item short form health survey	SF-36	36
Patient-reported outcomes measurement information system	PROMIS	121
Oswestry disability index	ODI	10
Roland–Morris questionnaire	RMQ	24
Zurich Claudication questionnaire	ZCQ	18
Scoliosis Research Society-22 Patient Questionnaire	SRS-22	22
Japanese orthopedic association back pain evaluation questionnaire	JOABPEQ	25
Western Ontario and McMaster Universities Arthritis Index	WOMAC	17
Knee Society scoring system	KSS	11
Bath ankylosing spondylitis functional index	BASFI	10
Health assessment questionnaire	HAQ	18
Pain disability assessment scale	PDAS	20
EuroQol 5-dimensions 5-levels	EQ5D-5L	5
Lumbar stiffness disability index	LSDI	10
The 25-question geriatric locomotive function scale	LOCOMO-25	25
Gastro-esophageal reflux disease questionnaire	GERDQ	6
Frequency scale for the symptoms of gastro-esophageal reflux disease	FSSG	12
	Total	390

In total, 390 items were placed into the following categories by content: (1) pain, (2) appearance, (3) sleeping, getting up from bed or floor and bedtime-related activities (4) sitting, standing up, and other sitting-related activities, (5) standing, walking, and stairs, (6) toilet and bathing-related activities, (7) dressing-related activities, (8) transportation, (10) housework, (11) sports, (12) social activities, (13) meals, and (14) mental health.

**Figure 3:**
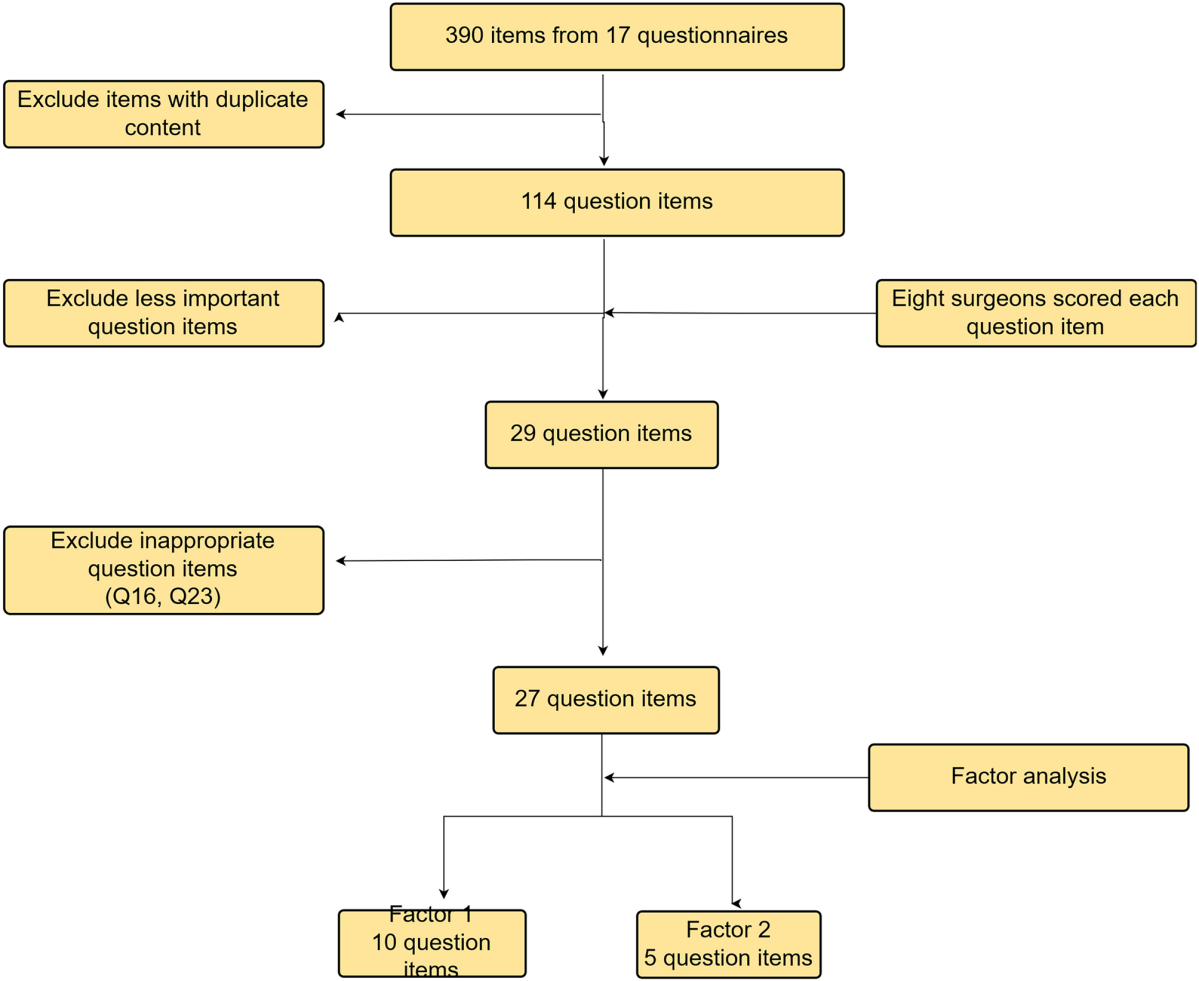
Flowchart of question item selection.

From these categories, we extracted 114 items that were considered useful for assessing ASD (Fig. 3). Sexual life, although an important item, was not included because of the expected large number of non-responses^28^. To ensure the relevance of questions to ASD in content validity, eight surgeons with extensive experience in operating on patients with ASD gave these 114 items a score from 3 to 0 according to their level of importance. We used the total score as a reference and selected 29 question items after discussion among the senior surgeons (Table 2). We modified detailed wording partially modified as appropriate. To examine the results comprehensibility, the developed questionnaire was given to three patients and one nurse, who reviewed the items in terms of text, meaning, and ambiguity and who provided feedback. Responses were on a five-point scale^29^, with an additional free-text field.

**Table 2:** Twenty-nine items for factor analysis selected after discussion among the surgeons.

Question item number	Content	Questionnaire	Answer options				
			Score point				
			1	2	3	4	5
1	Appearance	Are you concerned about the current appearance of your back?	Not at all	Not very much	Neither	Somewhat	Very much
2	Backpain	Which one of the following best describes the amount of back pain you have experienced?	No pain	A little pain	Moderate pain	Much pain	Severe pain
3	Leg pain	Which one of the following best describes the amount of buttock or leg pain you have experienced?	No pain	A little pain	Moderate pain	Much pain	Severe pain
4	Appetite	Do you have an appetite?	Yes, I have	A little	Neither	Not very much	None
5	Heartburn	In the last week, how many days have you had a burning sensation or burning pain in your chest?	Never	1 day	2 to 3 days	Between 4 and 6 days	7 days
6	Sleeping	Are you able to sleep on your back?	Can sleep without difficulty	Sometimes wake up	I have less than 6 h of sleep	I have less than 3 h of sleep	Cannot sleep
7	Standing up floors	Are you able to get up from the floor without help?	Can do easily	A little difficult	Somewhat difficult	Very difficult	Cannot do
8	Toilet	Are you able to wipe yourself after using the toilet?	Can do easily	A little difficult	Somewhat difficult	Very difficult	Cannot do
9	Picking up	Are you able to bend down and pick up something from the floor?	Can do easily	A little difficult	Somewhat difficult	Very difficult	Cannot do
10	Washing	Are you able to wash your body in the bath?	Can do easily	A little difficult	Somewhat difficult	Very difficult	Cannot do
11	Pants	Are you able to put on pants or trousers by yourself?	Can do easily	A little difficult	Somewhat difficult	Very difficult	Cannot do
12	Socks	Are you able to put on your socks?	Can do easily	A little difficult	Somewhat difficult	Very difficult	Cannot do
13	Sitting	Are you able to sit in a chair for a long time?	Can sit as long as I want	Can sit for 3 h	Can sit for 1 h	Can sit for 30 min	Can sit for 5 min
14	Standing up chairs	Are you able to stand up from a chair?	Can do easily	A little difficult	Somewhat difficult	Very difficult	Cannot do
15	Standing	How long can you keep standing without help?	Can stand more than 1 h	Can stand more than 30 min	Can stand more than 15 min	Can stand more than 5 min	Can stand more than one minute
16	Walking distance	How far can you keep walking without rest?	Can walk as far as I want	Can walk 1 km	Can walk 500 m	Can walk 300 m	Can walk 50 m
17	Walking time	How long can you keep walking without rest?	Can walk more than 1 h	Can walk more than 30 min	Can walk more than 15 min	Can walk more than 5 min	Can walk more than one minute
18	Stairs	Are you able to walk up and down the stairs?	Can do easily	A little difficult	Somewhat difficult	Very difficult	Cannot do
19	Ride	Are you able to get in and out of a car?	Can do easily	A little difficult	Somewhat difficult	Very difficult	Cannot do
20	Shelving	Are you able to reach to a high shelf?	Can do easily	A little difficult	Somewhat difficult	Very difficult	Cannot do
21	Hanging laundry	Are you able to hang your laundry on a clothesline?	Can do easily	A little difficult	Somewhat difficult	Very difficult	Cannot do
22	Light housework	Are you able to do simple tasks and housework (preparing meals, cleaning up, etc.)?	Can do easily	A little difficult	Somewhat difficult	Very difficult	Cannot do
23	Heavy housework	Are you able to do load-bearing tasks and housework (cleaning the yard, carrying heavy bedding, etc.)?	Can do easily	A little difficult	Somewhat difficult	Very difficult	Cannot do
24	Garbage	Are you able to take out the garbage?	Can do easily	A little difficult	Somewhat difficult	Very difficult	Cannot do
25	Dishwashing	Are you able to wash dishes, pots, and utensils by hand while standing at a sink?	Can do easily	A little difficult	Somewhat difficult	Very difficult	Cannot do
26	Sports	Are you able to play sports activity (jogging, swimming, gate ball, dancing, etc.)?	Can do easily	A little difficult	Somewhat difficult	Very difficult	Cannot do
27	Shopping	Are you able to carry objects weighting approximately 2 kg (2 standard milk bottles or 2 PET bottles each containing 1 L)?	Can do easily	A little difficult	Somewhat difficult	Very difficult	Cannot do
28	Community activity	Are you able to join social activities (meeting friends, playing sport, engaging in activities and hobbies, etc.)?	Can do easily	A little difficult	Somewhat difficult	Very difficult	Cannot do
29	Anxiety	Are you worried that you will not be able to walk in the future?	Not at all	A little anxious	Somewhat anxious	Fairly anxious	Very anxious

### Ethics statement

The study was conducted in accordance with the ethical standards of the Declaration of Helsinki. The study was approved by the local ethical review board (Osaka University Hospital Ethics Review Committee. No.11360). Written informed consent was obtained from each patient.

### Statistical analysis

The COSMIN guidelines introduce classical test theory and Rasch analysis for construct validation. We used classical test theory and factor analysis. Factor analysis was used to reduce and group the questions in order to create a valid, simple, and easy-to-use questionnaire. An exploratory factor analysis was performed using the maximum likelihood method on data from a total of 98 patients, including 89 postoperative responses and nine conservative cases. The number of factors was determined using the scree method. Because correlations between factors can be assumed, oblique rotation was performed using the Promax method. Finally, reliability was evaluated for content consistency using Cronbach’s coefficient alpha.

### Score calculation formula

Factor score coefficients obtained from factor analysis were used as a reference to correct the coefficients so that the scale’s total score ranged from 0 to 100. Specifically, individual items were weighted so that the difference between the minimum and maximum factor scores was approximately 100 depending on the choice of response^14^. However, we provided greater weight to those questions that clinicians deemed important. For example, 0 represented a limited health status and 100 represented an excellent health status.

### Comparison of scores and responsiveness

We compared the scores of the created scale, the PCS, and the MCS before and after surgery (paired t-test). Similarly, we compared the scale scores between the operated and conservative groups (unpaired t-test). We calculated Cohen’s d effect size by taking the difference between two means and dividing it by the standard deviation of the data. Cohen’s d effect size was used to evaluate the internal responsiveness of the scales. Next, we calculated Spearman’s correlation coefficients between the five satisfaction levels and the amount of score change on each scale. The external responsiveness of the scales was evaluated using Spearman’s correlation coefficients. An effect size of 0.2–0.49 was considered small, an effect size of 0.5–0.79 was considered moderate, and an effect size of 0.80 or greater was considered large^30^. A correlation coefficient of 0.2–0.39 was considered weak, a correlation coefficient of 0.4–0.69 was considered moderate, and a correlation coefficient of 0.70 or greater was considered strong. A p-value < 0.05 was considered statistically significant for two-tailed tests. SPSS Statistics (version 20; IBM, Armonk, NY, USA) was used for statistical analysis.

### External validation

We collected new patients with ASD from another institution for external validation. We applied our ASD disease-specific scale for these patients and compared the results with the internal validation data.

## Results

### Demographics of the patients

Of a total of 98 patients, 88 were women. The mean age of the 89 operative patients was 68 ± 7 years, and the mean time since the last surgery was 56 ± 35 months (Table 3). The mean number of fixed vertebral segments was 10 ± 3, including the sacrum or pelvis, in 76 patients (85%). The preoperative PCS was 31 ± 7 and improved to 41 ± 8 postoperatively (p < 0.0001). Postoperative satisfaction was 23 (26%) very satisfied, 42 (47%) satisfied, 18 (20%) neither satisfied nor dissatisfied, and 6 (7%) dissatisfied.

**Table 3:** Demographics of the study patients.

	Operative cases	Conservative cases
Male/female (cases)	10/79	0/9
Mean age ± SD (years)	68 ± 7	68 ± 12
Mean post operative follow-up period ± SD (mos.)	56 ± 35	N.A.
Mean fusion intervertebral levels ± SD	10 ± 3	N.A.
Fixation to sacrum or pelvis (cases/%)	76/85	N.A.
Fixation from T8, T9, or T10 to pelvis (cases/%)	56/63	N.A.
Fixation from T3, T4, or T5 to pelvis (cases/%) 13 / 15 0 / 0 0.01*	13/15	N.A.
Post operative satisfaction (cases/%)		
Very satisfied	23/26	N.A.
Satisfied	42/47	N.A.
Neither	18/20	N.A.
Dissatisfied	6/7	N.A.
Very dissatisfied	0/0	N.A.

*SD* standard deviation.

### Response of the patients

The results of the responses to each question are shown in Table 4, and the correlation coefficients are shown in Table 5. Seven patients had a free-text response of not performing Q23 heavy housework. Therefore, Q23 heavy housework was deemed inappropriate and excluded from the factor analysis. Regarding Q16 walking distance, four patients answered that they did not know the distance. Because there was a strong correlation between Q16 walking distance and Q17 walking time, we considered that Q17 walking time could be substituted for Q16 walking distance and excluded Q16. Factor analysis was conducted on the remaining 27 questions.

**Table 4:** Mean and standard deviation of raw data for each item.

Question item number	Content	Preoperative		Postoperative	
		Mean	SD	Mean	SD
1	Appearance	4.3	1.0	2.8	1.4
2	Backpain	3.6	1.4	2.1	0.9
3	Leg pain	2.9	1.4	2.1	0.9
4	Appetite	1.9	1.2	1.5	0.9
5	Heartburn	1.6	1.1	1.4	0.9
6	Sleeping	2.6	1.6	2.0	1.4
7	Standing up floors	2.2	1.1	2.6	1.2
8	Toilet	1.6	0.8	2.0	1.1
9	Picking up	2.0	1.1	2.8	1.3
10	Washing	1.8	1.0	2.1	1.1
11	Pants	1.8	0.9	2.4	1.0
12	Socks	2.0	1.2	2.8	1.2
13	Sitting	2.6	1.2	2.3	1.1
14	Standing up chairs	2.0	1.1	2.0	1.1
15	Standing	2.9	1.4	2.5	1.2
16	Walking distance	3.4	1.4	2.5	1.2
17	Walking time	2.7	1.1	2.1	1.0
18	Stairs	2.1	1.1	1.8	0.9
19	Ride	2.3	1.3	2.0	1.3
20	Shelving	3.1	1.3	2.4	1.2
21	Hanging laundry	2.6	1.3	2.0	1.3
22	Light housework	2.2	1.1	1.7	1.1
23	Heavy housework	2.7	1.3	2.4	1.3
24	Garbage	2.5	1.4	2.3	1.4
25	Dishwashing	2.5	1.2	1.7	1.1
26	Sports	4.0	1.3	3.8	1.3
27	Shopping	3.4	1.4	2.9	1.5
28	Community activity	3.6	1.5	3.1	1.5
29	Anxiety	3.8	1.3	2.7	1.4

Raw data means the score points of the answer options. For raw data, higher numbers indicate more activity restrictions.

*SD* standard deviation.

**Table 5:** Spearman correlation coefficients between each item for postoperative answers.

Question item number		1	2	3	4	5	6	7	8	9	10	11	12	13	14	15	16	17	18	19	20	21	22	23	24	25	26	27	28	29
		Appearance	Back pain	Leg pain	Appetite	Heartburn	Sleeping	Standing up floors	Toilet	Picking up	Washing	Pants	Socks	Sitting	Standing up chairs	Standing	Walking distance	Walking time	Stairs	Ride	Shelving	Hanging laundry	Light housework	Heavy housework	Garbage	Dishwashing	Sports	Shopping	Community activity	Anxiety
1	Appearance	1.0	0.43*	0.22*	0.04	0.21*	0.26*	0.08	− 0.11	0.05	0.08	0.07	− 0.05	0.23*	0.25*	0.20*	0.23*	0.34*	0.13	0.20*	0.36*	0.36*	0.31*	0.23*	0.16	0.28*	0.13	0.10	0.21*	0.40*
2	Back pain		1.0	0.41*	0.08	0.31*	0.32*	0.04	0.08	0.07	0.07	0.06	0.07	0.25*	0.25*	0.25*	0.31*	0.25*	0.05	0.24*	0.23*	0.23*	0.28*	0.23*	0.34*	0.24*	0.15	0.18	0.16	0.29*
3	leg Pain			1.0	0.16	0.29*	0.33*	0.25*	0.13	0.18	0.19	0.25*	0.19	0.17	0.43*	0.22*	0.29*	0.33*	0.30*	0.35*	0.37*	0.32*	0.35*	0.33*	0.32*	0.30*	0.22*	0.30*	0.34*	0.35*
4	Appetite				1.0	0.32*	0.17	0.14	0.16	0.15	0.19	0.13	0.08	0.17	0.04	0.06	0.23*	0.17	0.05	0.21*	0.15	0.13	0.31*	0.17	0.13	0.24*	0.25*	0.24*	0.12	0.08
5	Heartburn					1.0	0.35*	0.25*	0.16	0.23*	0.19	0.19	0.14	0.18	0.28*	0.25*	0.42*	0.46*	0.21*	0.27*	0.21*	0.34*	0.30*	0.16	0.22*	0.35*	0.11	0.24*	0.18	0.14
6	Sleeping						1.0	0.37*	0.32*	0.30*	0.15	0.24*	0.28*	0.30*	0.32*	0.28*	0.38*	0.37*	0.32*	0.40*	0.33*	0.23*	0.23*	0.30*	0.29*	0.25*	0.26*	0.35*	0.40*	0.29*
7	Standing up floors							1.0	0.38*	0.61*	0.42*	0.50*	0.52*	0.24*	0.46*	0.42*	0.42*	0.40*	0.45*	0.55*	0.36*	0.37*	0.38*	0.53*	0.41*	0.35*	0.59*	0.49*	0.59*	0.32*
8	Toilet								1.0	0.53*	0.58*	0.47*	0.61*	0.28*	0.22*	0.19	0.19	0.12	0.13	0.31*	0.19	0.19	0.24*	0.39*	0.27*	0.13	0.21*	0.23*	0.23*	0.17
9	Picking up									1.0	0.61*	0.68*	0.75*	0.36*	0.44*	0.38*	0.30*	0.36*	0.28*	0.50*	0.23*	0.43*	0.38*	0.54*	0.39*	0.34*	0.47*	0.40*	0.57*	0.25*
10	Washing										1.0	0.68*	0.61*	0.30*	0.33*	0.35*	0.24*	0.29*	0.24*	0.49*	0.29*	0.45*	0.52*	0.53*	0.39*	0.37*	0.31*	0.29*	0.38*	0.26*
11	Pants											1.0	0.76*	0.51*	0.51*	0.46*	0.39*	0.41*	0.32*	0.63*	0.34*	0.48*	0.48*	0.57*	0.47*	0.40*	0.45*	0.43*	0.48*	0.29*
12	Socks												1.0	0.37*	0.46*	0.40*	0.31*	0.35*	0.34*	0.49*	0.26*	0.36*	0.39*	0.50*	0.41*	0.31*	0.46*	0.44*	0.48*	0.25*
13	Sitting													1.0	0.40*	0.44*	0.45*	0.39*	0.27*	0.44*	0.35*	0.37*	0.43*	0.42*	0.41*	0.30*	0.36*	0.38*	0.35*	0.22*
14	Standing up chairs														1.0	0.60*	0.49*	0.59*	0.49*	0.68*	0.58*	0.64*	0.58*	0.61*	0.49*	0.61*	0.37*	0.49*	0.47*	0.52*
15	Standing															1.0	0.71*	0.76*	0.56*	0.69*	0.59*	0.62*	0.57*	0.62*	0.63*	0.57*	0.55*	0.51*	0.55*	0.47*
16	Walking distance																1.0	0.74*	0.51*	0.59*	0.54*	0.52*	0.50*	0.50*	0.54*	0.51*	0.58*	0.54*	0.59*	0.44*
17	Walking time																	1.0	0.49*	0.57*	0.52*	0.66*	0.56*	0.52*	0.49*	0.58*	0.50*	0.49*	0.56*	0.50*
18	Stairs																		1.0	0.52*	0.44*	0.52*	0.46*	0.39*	0.47*	0.52*	.450**	0.40*	0.57*	0.30*
19	Ride																			1.0	0.70*	0.70*	0.70*	0.72*	0.73*	0.68*	0.57*	0.61*	0.62*	0.56*
20	Shelving																				1.0	0.69*	0.65*	0.62*	0.66*	0.64*	0.41*	0.55*	0.55*	0.60*
21	Hanging laundry																					1.0	0.80*	0.64*	0.71*	0.81*	0.40*	0.50*	0.48*	0.55*
22	Light housework																						1.0	0.68*	0.64*	0.82*	0.46*	0.50*	0.42*	0.52*
23	Heavy housework																							1.0	0.69*	0.63*	0.53*	0.56*	0.51*	0.53*
24	Garbage																								1.0	0.68*	0.49*	0.61*	0.53*	0.46*
25	Dishwashing																									1.0	0.41*	0.53*	0.45*	0.49*
26	Sports																										1.0	0.67*	0.71*	0.45*
27	Shopping																											1.0	0.56*	0.49*
28	Community activity																												1.0	0.52*
29	Anxiety																													1.0

*Means p < 0.05.

### Factor analysis

The two-factor solution was adopted based on the decay status of the eigenvalues (scree criteria). The proportion of the total variance of the 27 items explained by the two factors before rotation was 47%.

Each item was ordered by factor loadings (Table 6). The first factor was named the main symptom because many of the symptoms were related to the patient’s primary complaints, such as the ability to do housework and walk, including Q25 dishwashing, Q21 laundry, Q20 shelving, and Q17 walking. The loadings for Q1 appearance, Q2 back pain, and Q29 anxiety were relatively low but were included because we considered these questions essential. We selected Q19 ride, Q24 garbage disposal, and Q15 standing as the remaining questions, according to factor loadings. Because Q22 light housework was strongly correlated with Q25 dishwashing (r = 0.82) and Q21 laundry (r = 0.80) and was considered to refer to the same thing, we excluded Q22. A total of 10 question items (Q1 appearance, Q2 back pain, Q15 standing, Q17 walking, Q19 ride, Q20 shelving, Q21 laundry, Q24 garbage disposal, Q25 dishwashing, Q29 anxiety) were used for the main symptom factor.

**Table 6:** Factor loadings and factor score coefficients.

Question item number	Content	Factor loading		Factor score coefficients	
		Factor 1	Factor 2	Factor 1	Factor 2
25	Dishwashing	0.941	− 0.069	0.195	− 0.011
21	Hanging laundry	0.888	− 0.011	0.161	0.008
20	Shelving	0.873	− 0.117	0.098	− 0.016
22	Light housework	0.869	0.027	0.165	0.020
17	Walking time	0.753	0.058	0.082	0.016
24	Garbage disposal	0.679	0.154	0.074	0.033
29	Anxiety	0.670	− 0.004	0.049	0.003
15	Standing	0.657	0.165	0.068	0.033
19	Ride	0.656	0.233	0.086	0.056
18	Stairs	0.610	0.105	0.047	0.017
14	Standing up chairs	0.512	0.240	0.041	0.034
27	Shopping	0.498	0.258	0.040	0.037
1	Appearance	0.479	− 0.260	0.022	− 0.019
5	Heartburn	0.453	0.003	0.023	0.002
2	Back pain	0.340	− 0.122	0.015	− 0.008
3	Leg pain	0.310	0.070	0.015	0.007
4	Appetite	0.207	0.063	0.009	0.005
12	Socks	− 0.230	1.009	− 0.028	0.294
9	Picking up	− 0.156	0.933	− 0.013	0.215
11	Pants	− 0.012	0.803	0.005	0.150
8	Toilet	− 0.157	0.667	− 0.007	0.068
10	Washing	0.041	0.652	0.006	0.083
7	Standing up floors	0.064	0.607	0.007	0.072
28	Community activity	0.308	0.469	0.028	0.066
26	Sports	0.286	0.419	0.022	0.050
13	Sitting	0.247	0.325	0.015	0.032
6	Sleeping	0.111	0.282	0.006	0.023

The second factor was named the collateral symptom because many items were related to postoperative limitation of movement, such as Q12 socks wearing and Q9 picking up. Because wearing Q11 pants and Q12 socks were highly correlated (r = 0.76), we excluded Q11 because Q12 socks could be substituted for Q11 pants. According to factor loadings, we selected five question items (Q7 standing up floors, Q8 toilet, Q9 picking up, Q10 washing, Q12 socks) as collateral symptom factors.

### Reliability

#### Internal consistency

The Cronbach’s alpha coefficient was 0.90 for the main symptom and 0.84 for the collateral symptom.

#### Calculation of scores

The factor score coefficients were used as weighting coefficients for each question, rounding the factor score coefficients to whole numbers to distribute the total scale score was distributed from 0 to 100. Because Q1 appearance and Q2 back pain are particularly important items, we gave them the same coefficients as Q25 dishwashing, which had a higher factor score coefficient. The better symptoms were set to 100 and the worse symptoms were set to 0. The calculation formulas are shown below (Supplement File 1).

1 $$ {\text{Main symptom score }}\left( {\text{first factor}} \right) \, = { 1}00 - ({\text{Q1}} \times {7 } + {\text{ Q2}} \times {7 } + {\text{ Q15}} \times {2 } + {\text{ Q17}} \times {3 } + {\text{ Q19}} \times {3 } + {\text{ Q 2}}0 \times {3} + {\text{ Q 21}} \times {6 } + {\text{ Q 24}} \times {3 } + {\text{ Q 25}} \times {7 } + {\text{ Q 29}} \times {2}) - {43})/{172} \times {1}00, $$

2 $$ {\text{Collateral symptom score }}\left( {\text{second factor}} \right) \, = { 1}00 - ({\text{Q 7}} \times {3} + {\text{ Q 8}} \times {3} + {\text{ Q 9}} \times {9} + {\text{ Q 1}}0 \times {3} + {\text{ Q 12}} \times {12}) - {3}0)/{15}0 \times {1}00. $$

### Score and responsiveness

#### Score change

The scores calculated based on the above formula are shown in Table 7. Comparing the operative and conservative groups, the main symptom of the operative group was 47 ± 21 preoperatively, while the conservative group was 63 ± 15. The operative group had significantly worse preoperative main symptoms than the conservative group (p = 0.029).

**Table 7:** Comparison of the final version scores between operative cases and conservative cases, and between preoperative condition and postoperative condition.

	Operative cases *N* = 89		Conservative cases *N* = 9		p-value (operative vs. conservative)	Effect size (operative vs. conservative)
Factor 1 Main symptom (10 questions)	Pre-operative condition	47 ± 21	Current condition	63 ± 15	0.029	0.77
	Post-operative condition	70 ± 22			0.3	0.35
	p-value (pre vs. post)	< 0.0001		N.A.	N.A.	
	Effect size (pre vs. post)	1.09				
Factor 2 Collateral symptom (5 questions)	Pre-operative condition	76 ± 25	Current condition	92 ± 12	0.005	0.67
	Post-operative condition	60 ± 25			0.0001	1.34
	p-value (pre vs. post)	0.0001		N.A.	N.A.	
	Effect size (pre vs. post)	0.65				
PCS (SF-8)	Pre-operative condition	31 ± 7	Current condition	35 ± 5	0.1	0.51
	Post-operative condition	41 ± 8			0.03	0.76
	p-value (pre vs. post)	0.0001		N.A.	N.A.	
	Effect size (pre vs. post)	1.26				
MCS (SF-8)	Pre-operative condition	43 ± 9	Current condition	47 ± 7	0.1	0.52
	Post-operative condition	49 ± 7			0.6	0.16
	p-value (pre vs. post)	0.0002		N.A.	N.A.	
	Effect size (pre vs. post)	0.40				

Scores are shown as mean ± SD.

*N.A.* not available, *SD* standard deviation.

However, the main symptom of the surgical group significantly improved to 70 ± 22 after surgery (p < 0.0001), exceeding those of the conservative group. As a result of the surgical improvement, there was no significant difference between the postoperative main symptom of the operative group and the main symptom of the conservative group (p = 0.3).

The mean collateral symptom score in the operative group worsened from 76 ± 25 preoperatively to 60 ± 25 postoperatively (p < 0.0001). The preoperative collateral symptom score in the operative group was significantly worse than that in the conservative group, 92 ± 12 (p = 0.005).

### Effect size

The effect size measured by Cohen’s d was 1.09, indicating a large effect size, for the main symptom for comparison of the preoperative and the postoperative score (Table 7). In the same comparison, the effect size of the collateral symptom was 0.65 (moderate), and that of the PCS was 1.26 (large).

In a comparison of operative and conservative groups, the effect size was 0.77 for the main symptom and 0.67 for the collateral symptom, indicating a moderate effect size.

#### Correlation coefficient

The Spearman’s correlation coefficient between satisfaction and the amount of score change was 0.48 (p < 0.001) for the main symptom and 0.38 for the PCS, both showing a moderate correlation (Table 8). The correlation coefficient between the main symptom and the PCS was 0.43, indicating a moderate correlation (p = 0.002).

**Table 8:** Spearman’s correlation coefficients between change scores and 5-point satisfaction rating scale.

	Satisfaction	Main symptom	Collateral symptom	PCS (SF-8)	MCS (SF-8)
Satisfaction	1	0.48*	0.16	0.38*	0.07
Main symptom		1	0.25*	0.43*	0.28*
Collateral symptom			1	0.11	0.22*
PCS (SF-8)				1	0.06
MCS (SF-8)					1

*Means *p* < 0.05.

#### Ceiling and floor effects

**Figure 4:**
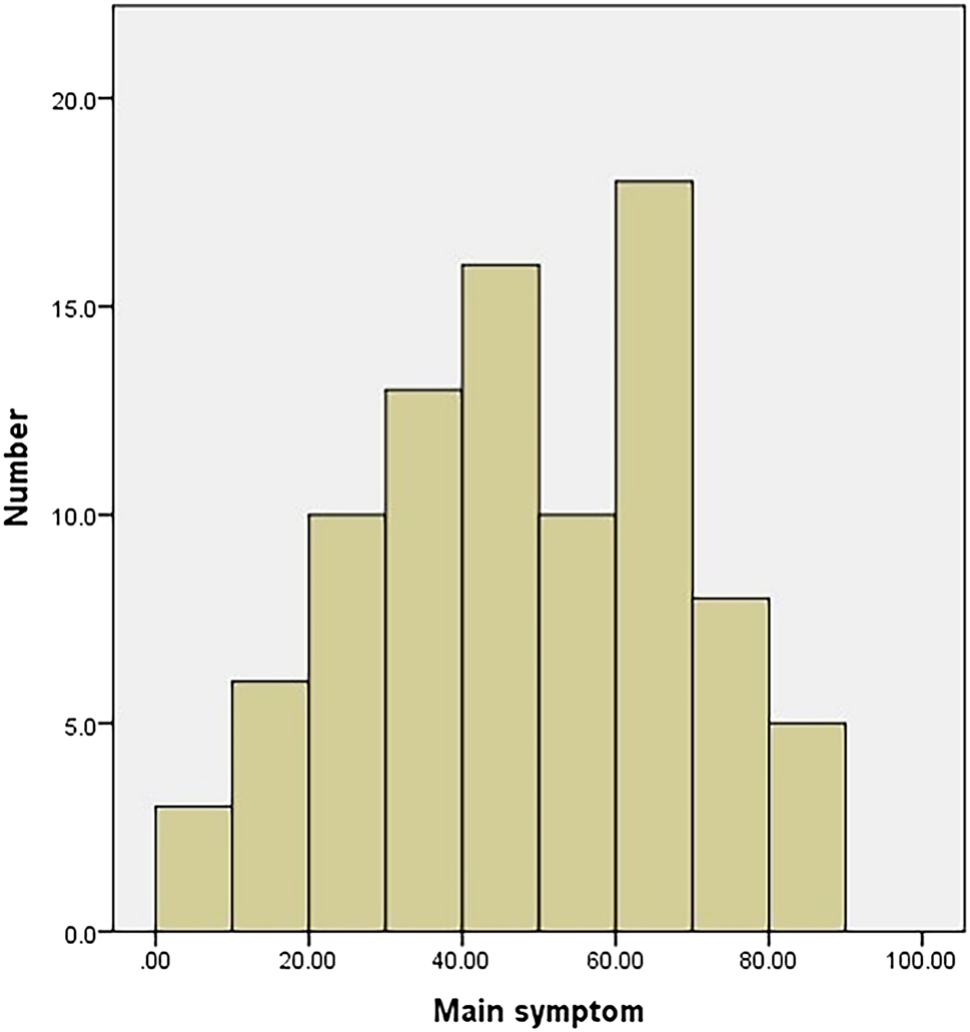
Histogram of the preoperative scores of the main symptom. The main symptom has no floor or ceiling effect.

**Figure 5:**
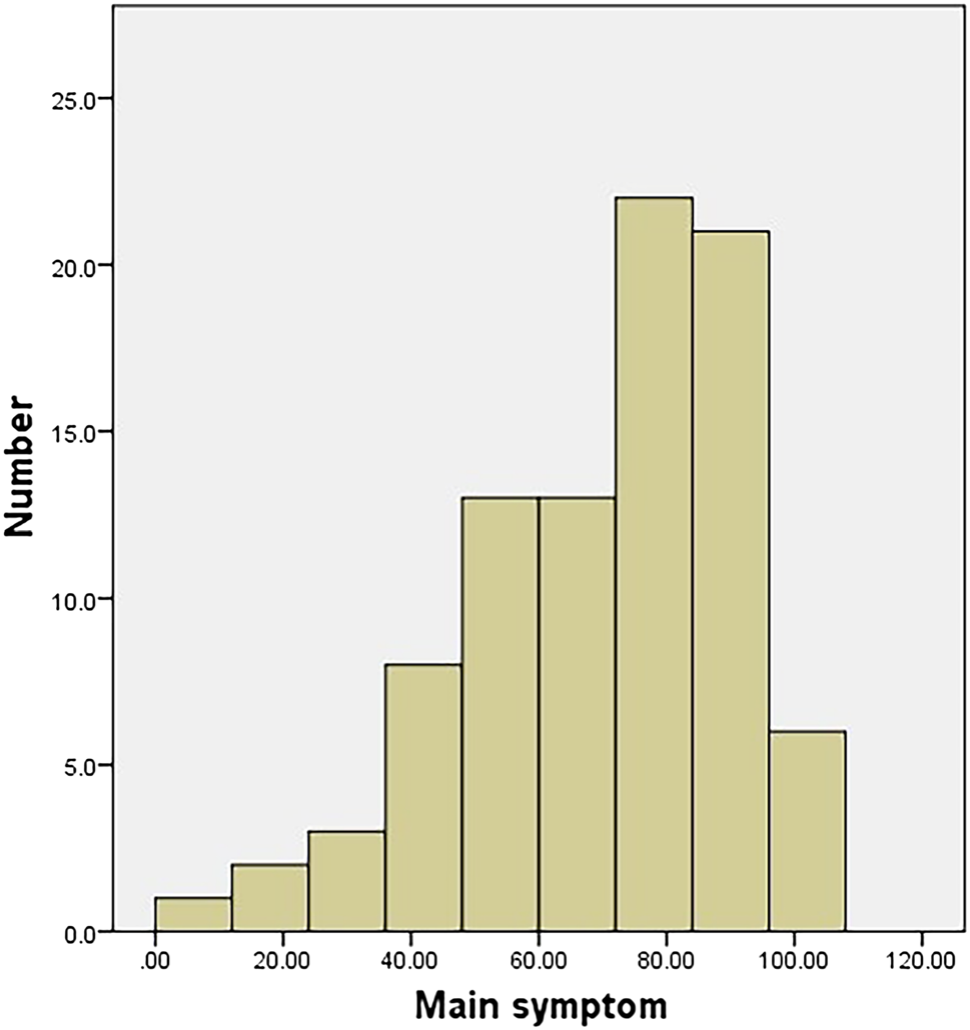
Histogram of the postoperative scores of the main symptom. The main symptom has no floor or ceiling effect.

**Figure 6:**
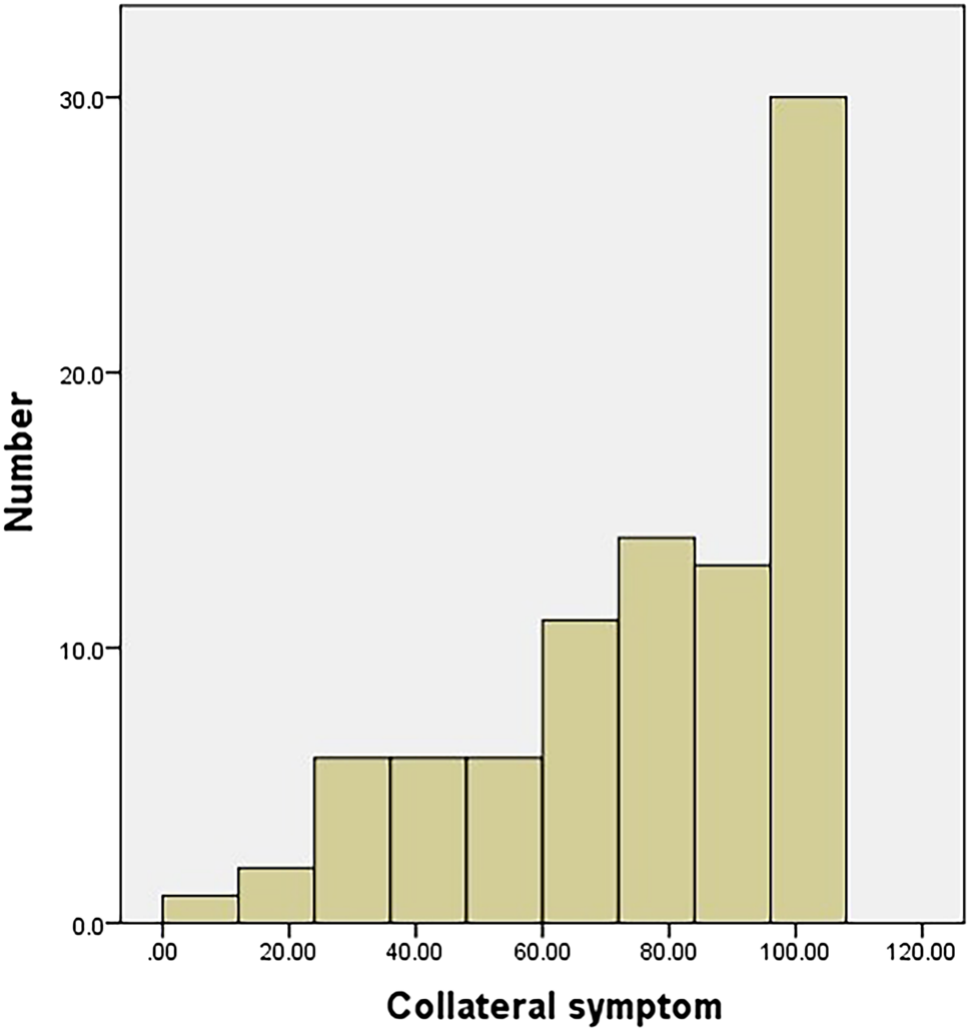
Histogram of the preoperative scores of the collateral symptom. The collateral symptom has no floor or ceiling effect.

**Figure 7:**
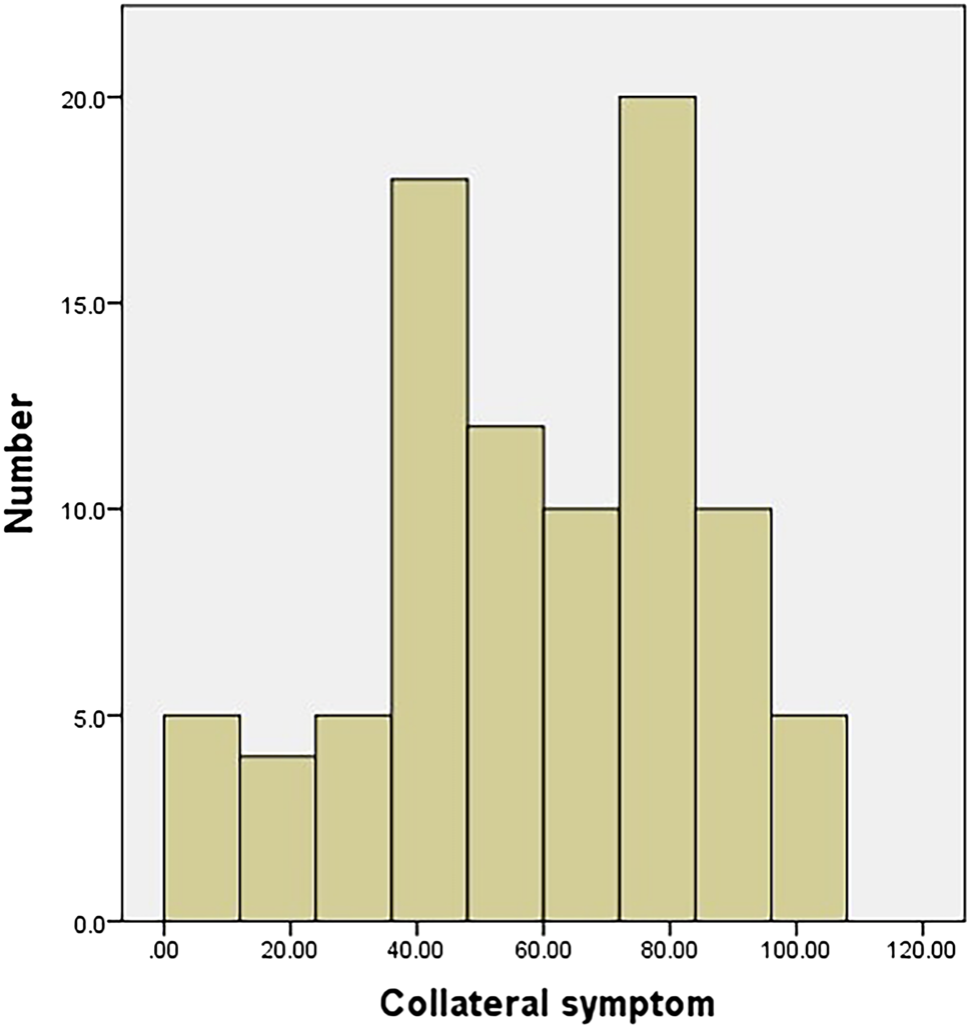
Histogram of the postoperative scores of the collateral symptom. The collateral symptom has no floor or ceiling effect.

The main symptoms had no floor or ceiling effect either preoperatively or postoperatively (Figs. 4, 5). Conversely, the collateral symptom had a ceiling effect preoperatively, but no floor effect postoperatively (Figs. 6, 7).

#### External validation

We added a new sample of 30 surgical patients with ASD in another facility for a disease-specific scale for ASD that we had created. This scale consisted of 10 main symptom and 5 collateral symptom questions, as described above. Total scores were calculated using the above formulas (Supplementary File 1). The SF-8 and satisfaction scale were enclosed, as well as the date when the scale was created.

Twenty-five people responded (Table 9). There was a significant difference in the age and fixation range between 25 patients for external validation and 89 patients for internal validation. However, no other background information was significantly different. The main symptom improved from 56 ± 19 preoperatively to 76 ± 19 postoperatively with an effect size of 1.05. The collateral symptom worsened from 75 ± 23 preoperatively to 64 ± 24 postoperatively with an effect size of 0.48. In both domains, the effect size was not different from the effect size at the time of scale creation, indicating the robustness of the scale.

**Table 9:** Validation data of the ASD disease-specific scale.

	Study subjects from two sites	Cases collected for validation from another facility	p-value
Male/female (cases)	10/79	2/23	1.0
Mean age ± SD (years)	68 ± 7	72 ± 8	0.02*
Mean post operative follow-up period ± SD (mos.)	56 ± 35	46 ± 22	0.1
Mean fusion intervertebral levels ± SD	10 ± 3	9 ± 1	0.1
Fixation to sacrum or pelvis (cases/%)	76/85	24/96	0.5
Fixation from T8, T9, or T10 to pelvis (cases/%)	56/63	25/100	0.01*
Fixation from T3, T4, or T5 to pelvis (cases/%) 13 / 15 0 / 0 0.01*		0/0	0.01*
Post operative satisfaction (cases/%)			
Very satisfied	23/26	8/32	0.6
Satisfied	42/47	11/44	0.8
Neither	18/20	5/20	1.0
Dissatisfied	6/7	1/4	1.0
Very dissatisfied	0/0	0/0	1.0
Main symptom (mean ± SD)			
Preoperative condition	47 ± 21	56 ± 19	0.05
Postoperative condition	70 ± 22	76 ± 19	0.2
Effect size	1.09	1.05	N.A.
Collateral symptom (mean ± SD)			
Preoperative condition	76 ± 25	75 ± 23	0.9
Postoperative condition	60 ± 25	64 ± 24	0.5
Effect size	0.65	0.48	N.A.
PCS (SF-8) (mean ± SD)			
Preoperative condition	31 ± 7	32 ± 7	0.6
Postoperative condition	41 ± 8	42 ± 8	0.4
Effect size	1.26	1.40	N.A.
MCS (SF-8) (mean ± SD)			
Preoperative condition	43 ± 9	41 ± 9	0.4
Postoperative condition	49 ± 7	46 ± 8	1.0
Effect size	0.40	0.56	N.A.

*ASD* adult spinal deformity, *N.A.* not available, *SD* standard deviation.

*Means *p* < 0.05.

## Discussion

This study is the first to use factor analysis to create a disease-specific scale for ASD. The most important point in a scale is to be able to measure the construct it is trying to measure^10,31^. Factor analysis is a technique used to explore and validate constructs, and is often used to create scales. “Intelligence” and “health” are examples of constructs that cannot be observed or measured directly. However, it is considered that they can be measured indirectly through multiple behaviors and events related to the construct^31^.

In the present study, factor analysis allowed us to detect two factors that constitute the construct of ASD. The first factor was named the main symptom because it reflected the patient’s main problems, such as appearance, pain, and housework activities. The second factor was named the collateral symptom, and was related to postoperative movement limitations such as putting on socks, picking up, and using the toilet. We considered that these two factors could measure the construct of ASD. The Cronbach’s alpha coefficients for each were 0.90 and 0.84, respectively, and had reliabilities that were acceptable for a clinically used measure.

In this study, the scale scores of the main symptom and the collateral symptom were calculated by weighting them according to the factor score coefficients. Both the main symptom and the collateral symptom showed significant differences in preoperative and postoperative comparisons of the surgery groups, and the effect size was large. Comparing preoperative scores of the surgery group and the conservative group also showed significant differences, and the effect size was moderate. In addition, the main symptom was significantly correlated with satisfaction and the PCS. These results indicated that the created scale had adequate responsiveness and criterion-related validity.

The items included in the factor analysis in this study were selected from various representative scales by physicians with extensive experience in ASD surgery and had content validity.

The SRS-22 is a commonly used outcome for assessing ASD, but several problems were noted^32^. Faraj et al. reviewed the strengths, weaknesses, and gaps of current outcomes in measuring ASD outcomes^33^. According to their study, the most frequently used outcome was the ODI, with the SRS-22s. However, they stated that both the ODI and the SRS-22 had weaknesses in their use to assess ASD. The ODI is a low back pain-specific questionnaire and does not necessarily include the concept of deformity. Conversely, the SRS-22 was developed for AIS, which is less functionally impaired and, therefore, is less relevant for ASD, which seeks to restore pain and quality of life. Faraj et al. stated that there was an overlap between the two outcomes and the need to develop a core outcome set that is more specific to the assessment of ASD.

Mannion et al. performed a factor analysis of the SRS-22 on ASD patients^34^. They found a poor fit for four questions on the SRS-22: Q3 (nervous person), Q14 (personal relationship), Q15 (financial difficulties), and Q17 (sick days). They recommended the deletion of these four questions.

Zaina et al. compared the newly developed Italian spine youth quality of life (ISYQOL) with the SRS-22 using Rasch analysis^35^. According to this group, Q15 (financial difficulties) in the SRS-22 was a poor fit, and they recommended 21 items except for that one. By excluding this item, the revised SRS-22 showed construct validity comparable with the ISYQOL.

Scheer et al. devised a patient generated index, a questionnaire that patients were asked to fill out freely^36^. The top 10 concerns of patients with ASD were walking, activities, posture, pain, sports, housework, relationships, gardening, sleeping, and traveling. The 29 items we selected almost covered these items. Of these items, about sports, some patients in this study indicated in their free-text sections that they did not engage in these activities. The term “sports” covers an extensive range, from light gymnastics and walking to running and swimming. We did not select Q26 sports because the factor loading was small and also because different people perceived this item differently.

Housework activities, conversely, are important for patients with ASD. In particular, as ASD is more common in older women, it is essential to include kitchen activities in the assessment. A kitchen elbow sign, for example, is a skin abnormality that develops on the elbow when working in the kitchen, as the patient must rest her elbow on a table to maintain a standing position^36^. In the current study, the factor loadings for washing dishes and laundry were large. Kitchen elbow sign is especially likely to occur when washing dishes because both hands are used, and the patient cannot hold a cane or walker during the task. Large factor loading of these two items suggests that patients with ASD have kyphosis, making it difficult for them to maintain an intermediate or dorsiflexed position.

Restriction of lumbar spine mobility after long fusion is a concern for both surgeons and patients^6^. Ishikawa et al. conducted a study about ADL for 36 long fusion patients^13^. They found that patients after long fusion performed better than preoperatively in activities such as sleeping supine, standing upright, vacuuming, doing laundry, and reaching for objects placed at heights. Conversely, strenuous activities such as shoveling snow worsened postoperatively. Overall surgical satisfaction was 70%. Their report suggests that long fusion surgery for ASD requires evaluating both positive and negative aspects.

Hart et al. investigated functional limitations due to lumbar stiffness in 62 patients^5^. They reported that 91% of the patients were satisfied with the trade-off between postoperative improvement in back pain and associated restriction of motion. In the present study, 73% of the patients were satisfied with their surgery. Their study included 24 cases (40%) of one vertebral fusion and only 19 cases (31%) of five or more vertebral fusion. Our patients had five or more intervertebral fusions, with an average of 10 fused vertebrae. This difference in fixation levels may have influenced the difference in satisfaction.

One of the advantages of our scoring system was that factor analysis divided the questions into two domains. The effect of surgery on ASD resulted in improved ADLs associated with improved pain and posture, but also movement limitations. Simply adding up these improvements and any worsening could result in a total score of plus or minus 0. By dividing this score into two domains, we could assess each symptom with each domain having the appropriate responsiveness. This represents two aspects of surgery for ASD and is a necessary component for improving treatment efficacy and explaining surgery to patients.

Another strength of this study was that the subject patients had an average of 10 long fixed vertebral intervertebral spaces, and 78% underwent fusion from the pelvic to the thoracic spine. Previous studies have focused on short lumbar intervertebral fusion procedures. Our patients are a more suitable population to assess ADLs for long fusion, especially as including L5/S in the fusion range would result in greater limitation.

There were some limitations in this study. The number of the patients was limited. Factor analysis was performed on 98 patients, slightly less than 100 patients. However, considering the two factors that were found, this could be considered sufficient. Because this study was conducted in one country, the results may not be generalizable to other countries. The burden of housework activities may differ between developed and developing countries. Reliability was assessed by content consistency, and a test–retest was not conducted in this study. The preoperative score was based on memory and there may have been recall bias. Patients with a longer follow-up period become more accustomed to their current symptoms and may underestimate the difference between their preoperative and current conditions. These issues should be addressed in future studies.

## Conclusion

We developed a disease-specific outcome for ASD using factor analysis. This analysis is the first scientifically validated measure that could simultaneously assess the benefits and limitations of ASD surgery. This tool can complement existing outcomes and will be useful for explaining surgery to patients and for future clinical trials.

### Supplementary Information

Supplementary Information.

## Data Availability

The datasets generated during and/or analyzed during the current study are available from the corresponding author on reasonable request.
